# Dogmatism

**Published:** 1888-05

**Authors:** 


### DOGMATISM.

It is astonishing how arrogantly and authoritatively some men will express themselves upon matters of which they are entirely ignorant. They get in the habit of doing a thing a certain way, and because that is the only manner with which they are acquainted, they loudly denounce every other method. We once heard a dentist declare that it was impossible to properly mix plaster of paris unless the plaster was added to the water instead of the water to the plaster. And yet, a little calm reflection should convince any one that it matters not which is placed in the bowl first. The only essential is thoroughly to incorporate the two so that the water of crystallization may be properly taken up by the dry powder.

We once heard an excellent operator dogmatically declare that in no case was it necessary, or even expedient, to place the rubber dam over more than two teeth in filling any cavity—that to include more was a wicked waste of time, dam, patient and operator. He admitted that he had never tried more—did not need to; he could succeed perfectly without. If a dentist will try the plan of including two teeth on each side of the cavity to be filled, he must be convinced that there are times when it will greatly add to his convenience and the perfection of the operation.

Nothing will convince some men that there can be a better way than their own. A dentist once informed us that there was no implement fit to mix plaster with except a tablespoon, and to sustain his position he said that a spoon was naturally intended for mixing purposes, for the concavity of the bowl produced rotary currents in the mass to be mixed, which insured a thorough commingling of the particles. When reminded that a spoon was inconvenient for building up the plaster, he said he could do that well enough with his fingers. In dental society meetings this dogmatic assurance is constantly bubbling up, and men who, perhaps, are the least qualified for the task, deliberately set about the instruction of their colleagues in the most rudimental of technics, dwelling upon particular methods to the total exclusion of principles and mechanical laws, and upon mere methods to an end waste time which should be devoted to a better purpose.

